# UDP-glucose dehydrogenase modulates proteoglycan synthesis in articular chondrocytes: its possible involvement and regulation in osteoarthritis

**DOI:** 10.1186/s13075-014-0484-2

**Published:** 2014-12-03

**Authors:** Yinxian Wen, Jing Li, Linlong Wang, Kai Tie, Jacques Magdalou, Liaobin Chen, Hui Wang

**Affiliations:** Department of Orthopedic Surgery, Zhongnan Hospital of Wuhan University, Wuhan, 430071 China; UMR 7365 CNRS-Nancy Université, Faculté de Médecine, Vandoeuvre-lès-Nancy, France; Department of pharmacology, School of Basic Medical Science, Wuhan University, Wuhan, 430071 China; Hubei Provincial Key Laboratory of Developmentally Originated Disease, Wuhan, 430071 China

## Abstract

**Introduction:**

The objective of this study was to investigate the possible role of UDP-glucose dehydrogenase (*UGDH*) in osteoarthritis (OA) and uncover whether, furthermore how interleukin-1beta (IL-1β) affects *UGDH* gene expression.

**Methods:**

*UGDH* specific siRNAs were applied to determine the role of *UGDH* in proteoglycan (PG) synthesis in human articular chondrocytes. Protein levels of *UGDH* and *Sp1* in human and rat OA cartilage were detected. Then, human primary chondrocytes were treated with IL-1β to find out whether and how IL-1β could regulate the gene expression of *UGDH* and its trans-regulators, that is *Sp1*, *Sp3* and *c-Krox*. Finally, p38 mitogen-activated protein kinase (MAPK) inhibitor SB203580 and stress-activated protein kinase/c-Jun N-terminal kinase (SAP/JNK) inhibitor SP600125 were used to pick out the pathway that mediated the IL-1β-modulated PGs synthesis and gene expression of *UGDH*, *Sp1*, *Sp3* and *c-Krox*.

**Results:**

*UGDH* specific siRNAs markedly inhibited *UGDH* mRNA and protein expression, and thus led to an obvious suppression of PGs synthesis in human articular chondrocytes. *UGDH* protein level in human and rat OA cartilage were much lower than the corresponding controls and negatively correlated to the degree of OA. Decrease in *Sp1* protein level was also observed in human and rat OA cartilage respectively. Meanwhile, IL-1β suppressed *UGDH* gene expression in human articular chondrocytes in the late phase, which also modulated gene expression of *Sp1*, *Sp3* and *c-Krox* and increased both *Sp3*/*Sp1* and *c-Krox*/*Sp1* ratio. Moreover, the inhibition of SAP/JNK and p38 MAPK pathways both resulted in an obvious attenuation of the IL-1β-induced suppression on the UGDH gene expression.

**Conclusions:**

UGDH is essential in the PGs synthesis of articular chondrocytes, while the suppressed expression of *UGDH* might probably be involved in advanced OA, partly due to the modulation of p38 MAPK and SAP/JNK pathways and its trans-regulators by IL-1β.

**Electronic supplementary material:**

The online version of this article (doi:10.1186/s13075-014-0484-2) contains supplementary material, which is available to authorized users.

## Introduction

Proteoglycans (PGs) are glycoconjugates composed of a core protein backbone and numerous glycosaminoglycan (GAG) side chains, which determine the fluid and electrolyte balance and the elasticity of articular cartilage and provide the living space for chondrocytes through interaction with the collagen network. Thus, PGs are essential in maintaining cartilage homeostasis [1]. Loss of PGs would lead to the imbalance of cartilage homeostasis, which further accelerates the degeneration of cartilage matrix and the apoptosis of chondrocytes, and finally triggers the pathogenesis of osteoarthritis (OA), a chronic and degenerative arthritis with a high prevalence in the elderly [1,2].

UDP-glucose dehydrogenase (UGDH) catalyzes the transformation of UDP-glucose to UDP-glucuronic acid, a key precursor for the synthesis of the GAG chain in PGs [3-6]. Stimulating UGDH enzyme activity with transforming growth factor β (TGF-β) resulted in the enhanced GAG synthesis in articular chondrocytes [7]. However, whether *UGDH* is indispensable in the PG synthesis of articular chondrocytes and whether *UGDH* is also involved in the pathogenesis of OA still remain unclear.

IL-1β is a key pro-inflammatory cytokine in the progression of OA, which attenuates the anabolism but enhances the catabolism in the chondrocytes, through activating the downstream signaling pathways, including those of stress-activated protein kinase/c-Jun N-terminal kinase (SAP/JNK) and p38 mitogen-activated protein kinase (p38 MAPK) [8-10]. It is well-known that IL-1β is one of the key pro-inflammatory factors responsible for the PG loss in OA pathogenesis. However, whether *UGDH* is involved in the IL-1β-induced PG loss is unknown.

Specificity protein 1 (*Sp1*), *Sp3* and Krueppel-related zinc finger protein cKrox (*c-Krox*) are trans-regulators sharing almost the same binding sites located in the promoter region of *UGDH* gene [11,12]. *Sp1* recognizes GC- or GT-rich motifs and presents positive regulatory effects on the transcriptional activity of the *UGDH* gene [13,14]. *Sp3* is another member of the *Sp* family, which represses *Sp1*-mediated activation of gene transcription due to the competition for their common binding sites [12]. Meanwhile, *c-Krox*, the key trans-regulator of type 1 collagen [15], inhibits gene transcription of *UGDH* in chondrocytes [11].

So, we hypothesized that *UGDH* is essential in the PG synthesis of articular chondrocytes, and that IL-1β inhibits *UGDH* gene expression through modulating *UGDH* trans-regulators and the downstream signaling cascades, including the SAP/JNK and p38 MAPK pathways, which might be involved in the PGs loss of OA cartilage and contribute to the OA pathogenesis. So, we detected PG content in human primary chondrocytes treated with *UGDH*-specific siRNA, measured the protein level of *UGDH* and *Sp1* in human and rat OA cartilage and detected the influence of the activation and inhibition of SAP/JNK or p38 MAPK pathways on the gene expression of *UGDH* and its trans-regulators in human articular chondrocytes, in an attempt to uncover the role of *UGDH* in the PG synthesis of articular chondrocytes and the pathogenesis of OA.

## Methods

### Cartilage specimens

Human articular cartilage specimens from the knee joints were obtained from OA patients diagnosed with advanced OA using the criteria of the American College of Rheumatology for OA undergoing total knee replacement surgery (21 knees from 15 female patients, aged 66 ± 10 years) with signed informed consent [16]. The procedures were in accordance to the ethical guidelines of the Helsinki Declaration of 1975 (as revised in 2000) and approved by Medical Ethics Committee of the Zhongnan Hospital of Wuhan University (number 2012030). Microscopically normal cartilage (MNC) and degenerative cartilage (DC) from the same patient was collected respectively from the tibial plateau using a surgical microscope with 8-fold amplification, paired and numbered [17].

Pathogen-free adult Wistar rats (weight 220 to 280 g) were supplied by Experimental Centre of Medical Scientific Academy of Hubei province, which also approved the animal study protocol applied in the study (number 2008–0005). The protocol was in accordance with the Guide for the Care and Use of Laboratory Animals (eighth edition) by the National Research Council of the United States National Academies. The animal study was performed in the Animal Biosafety Level-3 Laboratory of Wuhan University (Wuhan, China) accredited by the Association for Assessment and Accreditation of Laboratory Animal Care International (AAALAC). The OA model was induced as described previously [18]. Sixteen rats were grouped into the control group and the OA group, which were intra-articularly injected respectively with 20 μL of sterile 0.9% saline or 4% (w/v) papain (Sigma-Aldrich, MO, USA) solution in saline to the right knees of the rats on days 1, 4 and 7. Two weeks after the last injection, all the rats were sacrificed under anesthesia for the knee joints.

### Histopathology assay

Cartilage samples from the weight-bearing area of the knee joint were used in pathological testing. Human MNC samples were defined as the control, while the DC samples were defined as the OA cartilage. Samples of human and rat cartilage were fixed in 4% paraformaldehyde overnight and embedded in paraffin wax, successively. Then, sections of 5 μm were obtained perpendicularly to the surface of articular cartilage. HE and Safranin O staining was performed according to the standard protocol. The degree of OA was presented independently by three observers according to the modified Mankin scoring system using a blinded method [19]. Moreover, protein expression of UGDH and Sp1 in the chondrocytes was also detected using immunohistochemical (IHC) assay with anti-UGDH (1:150, Proteintech, Chicago, IL, USA) and anti-Sp1 (1:150, Proteintech) antibodies. The relative protein level of UGDH and Sp1 was presented as the mean absorbance of each positively stained chondrocyte using NIS-elements software (Nikon, Tokyo, Japan).

### Chondrocyte isolation, culture and treatment

Human cartilage samples without microscopically visible degeneration were dissected and digested with 0.25% trypsin (Sigma-Aldrich, MO, USA) for 30 minutes and 0.2% collagenase typeII (Sigma-Aldrich) for 12 h in serum-free DMEM/F-12 (1:1 v/v) (Thermo Fisher, Beijing, China). Then, chondrocytes were collected and cultured as a monolayer in DMEM/F12 (1:1 v/v) with 10% (v/v) fetal bovine serum (Thermo Fisher), 100 IU/mL penicillin (Biyotime, Haimen, China), 100 μg/mL streptomycin (Biyotime), and 2 mM glutamine (Biyotime) at 37°C with 5% CO_2_. Hereafter, the chondrocytes were treated with *UGDH*-specific siRNA for 4 h using Lipofectamine 2000 Reagent (Life technologies, Grand Island, NY, USA) and cultured for another 48 h following the manufacturer’s protocol. Details of the *UGDH*-specific siRNA are listed in Table 1. Chondrocytes were also treated with human recombinant IL-1β (10, 20, 40 ng/mL, PeproTech, Rocky Hill, NJ, USA) for 12, 24 and 48 h, and were also pretreated with p38 MAPK inhibitor SB203580 (20 μM, Sigma-Aldrich) or SAP/JNK inhibitor SP600125 (10 μM, Sigma-Aldrich) for 0.5 h and subsequently co-treated with 10 ng/mL IL-1β for another 48 h, to detect the mRNA and protein level of the genes of interest. Meanwhile, chondrocytes were also treated with IL-1β (10 ng/mL) for 0 to 120 minutes or pretreated with SP600125 or SB203580 for 30 miutes and then treated with 10 ng/mL IL-1β for another 30 minutes for the phosphorylation status of JNK and p38 MAPK. Chondrocytes from at least three individuals were applied in every *in vitro* experiment.

**Table 1:** **The small interfering RNA applied in the study**

**Target genes**	**Gene ID**	**siRNA pairs**	**Sequence**
*UGDH*	2582	Pair 1	F: 5′-CUGAGUGGGACAUGUUUAATT -3′
			R: 5′-UUAAACAUGUCCCACUCAGTT -3′
		Pair 2	F: 5′-CAGCCAUCAAGGACCUAAATT -3′
			R: 5′-UUUAGGUCCUUGAUGGCUGTT -3′
		Pair 3	F: 5′-GCCAGAAGUAGCUCGUUAUTT -3′
			R: 5′-AUAACGAGCUACUUCUGGCTT -3′
Negative control	NA	Pair 1	F: 5′-UUCUCCGAACGUGUCACGUTT-3′
			R: 5′-ACTUGACACGUUCGGAGAATT-3′

*UGDH*, UDP-glucose dehydrogenase; NA, not applicable.

### GAG detection

GAG content was detected using 1,9-dimethylmethylene blue (DMB, Sigma-Aldrich) reagent as reported [20-22]. Absorbance at 570 nm was measured using a UV-1601 spectrophotometer (Shimadzu, Kyoto, Japan). A standard curve constructed with chondroitin sulfate sodium salt from shark cartilage (Sigma-Aldrich) was used to quantify GAG content in the chondrocyte cultures. Then, total GAG was determined as GAG content versus protein content of the same culture. Meanwhile, chondrocytes were cultured on coverslips, fixed in 10% (w/v) neutral formalin for 15 minutes, stained with 0.5% (w/v) Alcian blue dye and photographed using an AZ100 Microscopes (Nikon, Tokyo, Japan). Relative GAG content was determined as mean absorbance of each positively stained chondrocyte.

### Real-time quantitative PCR assay

Real-time quantitative PCR assay was performed as previously described [23]. Total RNA was isolated using TRIzol reagent (Life Technologies). Single-strand cDNA was obtained using a First Strand cDNA Synthesis Kit (Takara, Dalian, China). Primer Premier 5.0 (Premier Biosoft, Palo Alto, CA, USA) and the NCBI BLAST database were applied to design the primers for the genes of interest. The primers used in this study are listed in Table 2. The RT-PCR assay was performed on a StepOne thermal cycler (Applied Biosystems, Grand Island, NY, USA) using reverse-transcription (RT)-PCR kits (Takara) using the following procedure: pre-denaturation at 95°C for 30 sec, denaturation at 95°C for 5 sec, annealing at T_m_ for 30 sec, and extension at 72°C for 30 sec. The last three steps ran for 40 cycles. Relative standard curves were constructed for relative quantification. The expression of all the target genes was normalized to the *GAPDH* gene to standardize comparison.

**Table 2:** **The primers used in the study**

**Target genes**	**Gene ID**	**Sequence**	**Annealing (°C)**	**Product size (bp)**
*UGDH*	2582	F: 5′-CAGGCTATGTTGGAGGACCC-3′	60	162
		R: 5′-TCGACAGGATTCTACCACTTCTT-3′		
*Sp1*	6667	F: 5′-ATGGACAGGTCAGTTGGCAG-3′	60	89
		R: 5′-CTGCATTGGGGCTAAGGTGA-3′		
*Sp3*	6670	F: 5′-CAGTCAGCAGATGGTCAGCA-3′	60	185
		R: 5′-CCCTGAACCTGGACTTGACC-3′		
*c-Krox*	51043	F: 5′-CGGTGTTCGATTCACCAGGA-3′	60	134
		R:5′-GCAGGTGCATGTGGTTCTTG-3′		
*GAPDH*	2597	F: 5′-GAAATCCCATCACCATCTTCCAG-3′	60	313
		R:5′-GAGTCCTTCCACGATACCAAAG-3′		

*UGDH*, UDP-glucose dehydrogenase; *Sp1*, specificity protein 1; *Sp3*, specificity protein 3; *c-Krox*, krueppel-related zinc finger protein cKrox; *GAPDH*, glyceraldehyde-3-phosphate dehydrogenase.

### Western blotting assay

Total proteins were obtained from human cartilage samples and chondrocyte cultures using radioimmunoprecipitation assay (RIPA) lysis buffer (Biyotime), while nuclear proteins were extracted using a nuclear protein extraction kit (Biyotime). Then, proteins were size-fractionated by SDS-PAGE and transferred to nitrocellulose membranes (Millipore, Bellerica, MA, USA). The target proteins were probed with anti-UGDH (1:1,000, Proteintech), anti-Sp1 (1:800, Proteintech), anti-Phospho-SAPK/JNK (Tyr185) (1:500, Enogene, Nanjing, China), anti-Phospho-p38 MAPK (Thr180) (1:500, Enogene), anti-SAPK/JNK (1:1000, Sangon, Shanghai, China), anti-p38 MAPK (1:1000, Sangon), anti-GAPDH (1:1,000, Proteintech) and anti-lamin A/C (1:1,000, Proteintech) primary antibodies, incubated with horseradish peroxidase-conjugated secondary antibody (1:5,000, Proteintech). Blots were developed using ECL reagent (Advansta, Menlo Park CA, USA). A Fusion FX system (Vilber Lourmat, Marne-la-Vallée, France) was applied to photograph the blots. Then, relative the protein level of UGDH and SP1 was obtained using Quantity One software (Version 4.6, Bio-Rad, Berkeley, CA, USA), compared with the corresponding controls and standardized to GAPDH.

### Statistical analysis

Data analysis was performed using SPSS 17.0 (SPSS Science Inc., Armonk, NY, USA) and Prism 5.0 (GraphPad Software, San Diego, CA, USA). Results were presented as mean ± standard error of the mean (SEM). One-way analysis of variance (ANOVA) or Student’s *t*-test, as appropriate, after testing the homogeneity of variances, were performed to analyze the data. The Wilcoxon rank-sum test was applied to analyze the difference between Mankin scores for cartilage from the control and the OA group. Spearman rank correlation analysis was performed to test the correlation between Mankin score and UGDH protein level in human and rat cartilage. Values of *P* <0.05 were considered statistically significant.

## Results

### UGDH was essential in PG synthesis of human articular chondrocytes

**Figure 1:**
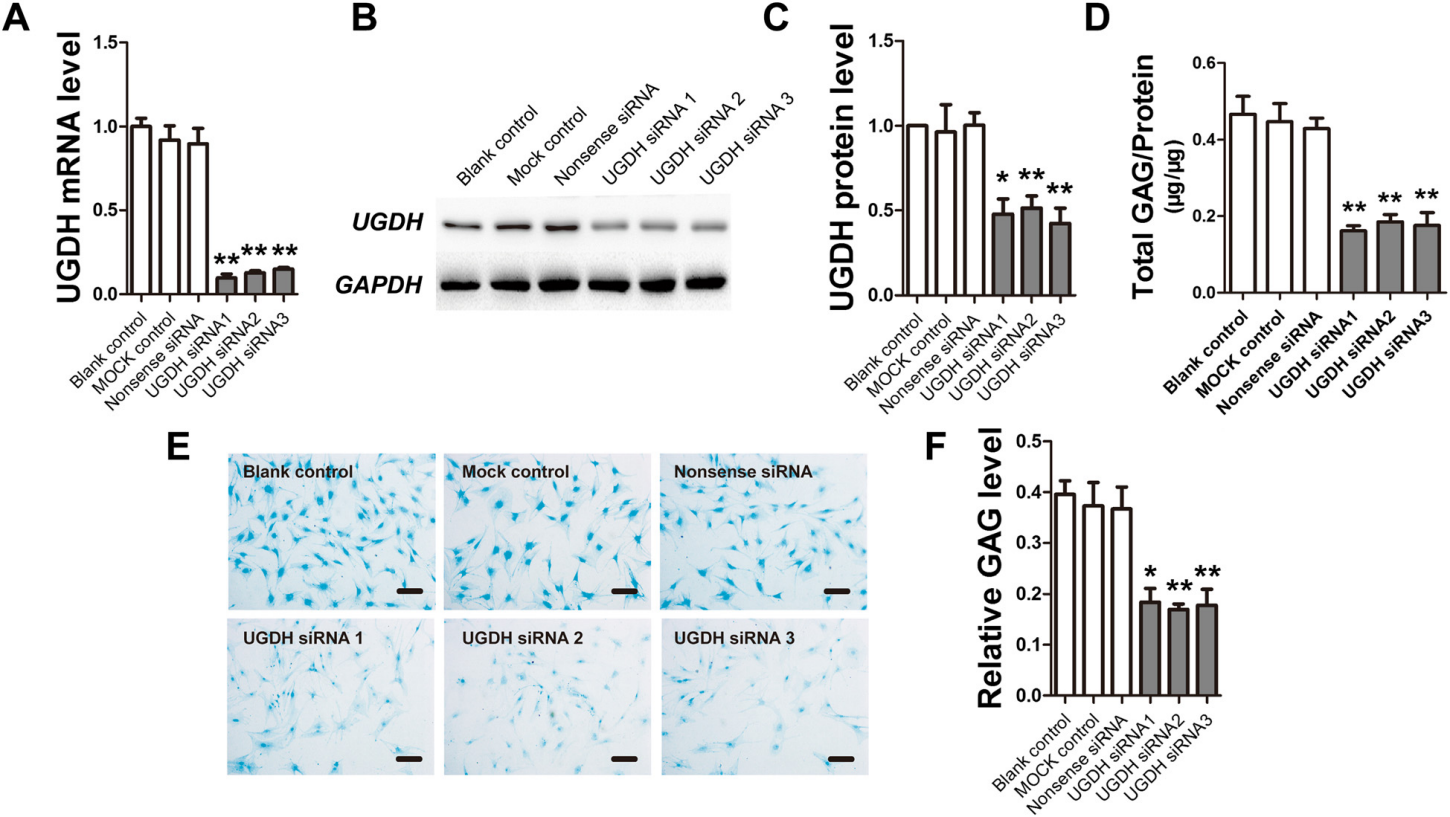
**UDP-glucose dehydrogenase (** ***UGDH*** **)-specific siRNA suppressed glycosaminoglycan (GAG) synthesis in human articular chondrocytes.** Human articular chondrocytes were treated with *UGDH*-specific siRNA for 48 h. Then, mRNA and the protein level of *UGDH* were detected using real-time PCR **(A)** and western blotting assay **(B, C)**, while GAG content of these chondrocyte cultures were detected with 1,9-dimethylmethylene blue dye **(D)** and Alcian blue dye **(E, F)**. The protein level of *UGDH* was presented as gray level of the blots quantified using Quantity One software (Version 4.6, Bio-Rad). Values are presented as mean ± standard error of the mean from at least three independent experiments. *P <0.05 and **P <0.01 versus control.

Obvious decreases in *UGDH* mRNA and protein levels were observed in human articular chondrocytes treated with three different *UGDH*-specific siRNA (Figure 1A, B and C, *P* <0.05), which was accompanied by a decrease in total GAG content in the chondrocyte cultures (Figure 1D, *P* <0.05). Meanwhile, the staining of chondrocytes treated with *UGDH*-specific siRNA by Alcian blue was much lower than the control (Figure 1E and F, *P* <0.05), which also indicated the suppression of PG synthesis in the chondrocytes due to inhibited *UGDH* gene expression.

### UGDH protein expression was decreased in OA cartilage

**Figure 2:**
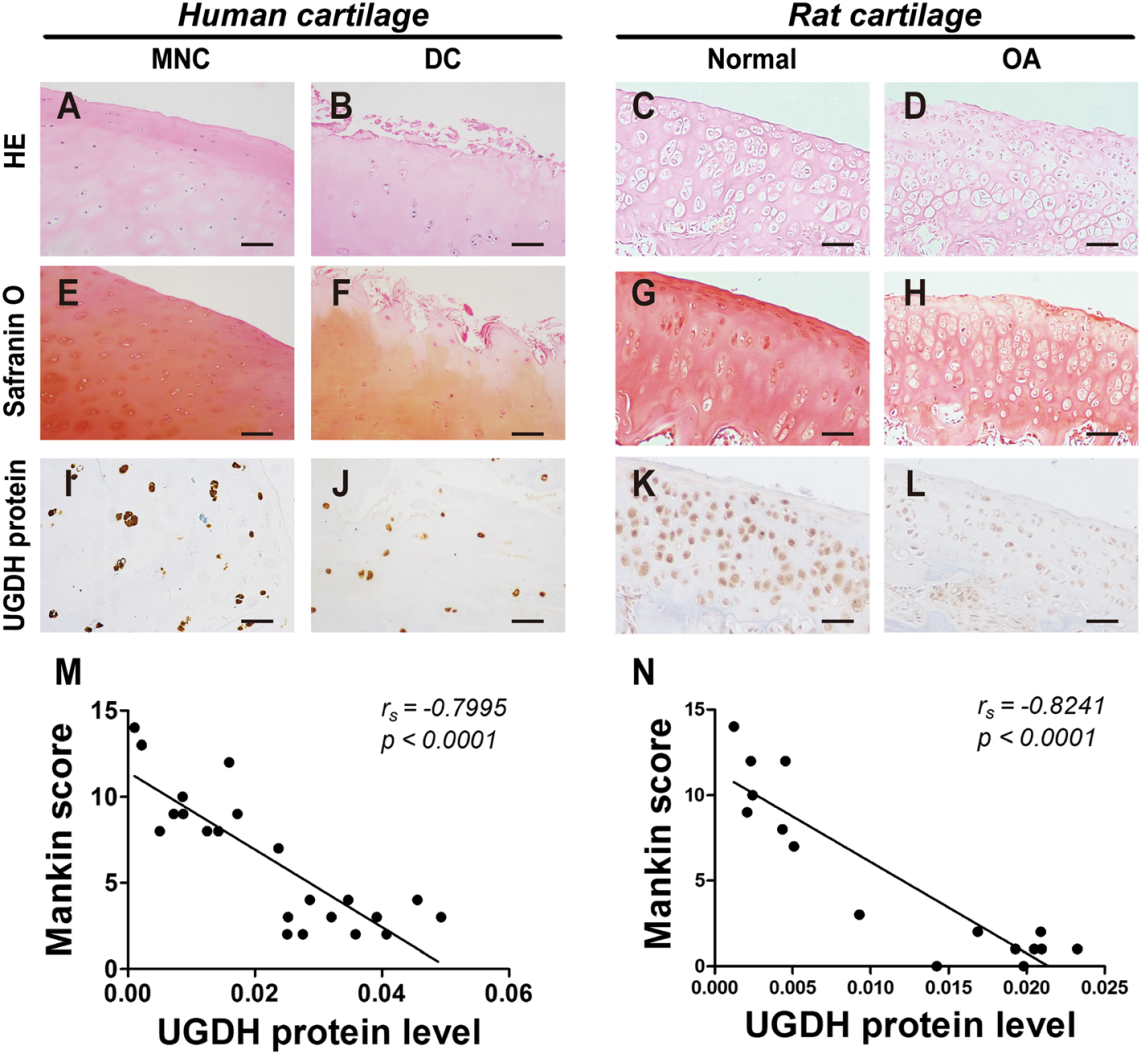
**Histopathology assay of human and rat osteoarthritis (OA) cartilage.** Human cartilage from the tibial plateau of OA patients was collected, and the rat OA model was induced. Human OA cartilage was divided as microscopically normal cartilage (MNC) and degenerative cartilage (DC). The former was defined as the control, and the latter as OA cartilage. HE staining **(A-D)** and Safranin O staining **(E-H)** was performed. UDP-glucose dehydrogenase (UGDH) protein level was detected using immunohistochemical (IHC) assay **(I, L)** and presented as mean optical density of each chondrocytes using NIS-Elements software (Nikon, Tokyo, Japan), and the modified Mankin score system was applied to grade the degree of OA. Cartilage samples from 11 knees were included in the pathological analysis. Then, correlation between the Mankin score and UGDH protein level of human and rat cartilage **(M, N)** was performed. Scale bars, 100 μm.

Serious degenerative features of human OA cartilage, namely extensive surface irregularities, clefts to calcified zone, and even complete disorganization, were observed using HE staining (Figure 2A and B). A marked decrease in GAG content was observed in DC by safranin O staining, when compared with MNC from the same OA patient (Figure 2E and F). Mankin scores of MNC were also much lower than those for DC (*P* <0.05), while the UGDH protein level of DC was also significantly lower than that of MNC (*P* <0.05). An additional figure file shows this in more detail (see Additional file 1A). Similar degenerative features were also observed in rat OA cartilage, together with an increase of chondrocyte numbers (Figure 2C and D). Safranin O staining of rat OA cartilage was also much lighter (Figure 2G and H). Moreover, Mankin scores for rat OA cartilage were much higher (*P* <0.05), while UGDH protein level was also lower when compared with normal control (*P* <0.05). An additional figure file shows this in more detail (see Additional file 1B). Further correlation analysis showed that UGDH protein level in both human and rat cartilage was negatively correlated with the Mankin score (*P* <0.05, Figure 2M and N), which indicated a strong correlation between the suppressed *UGDH* protein level with the stimulated cartilage degeneration during OA.

### IL-1β decreased UGDH gene expression and inhibited GAG synthesis

**Figure 3:**
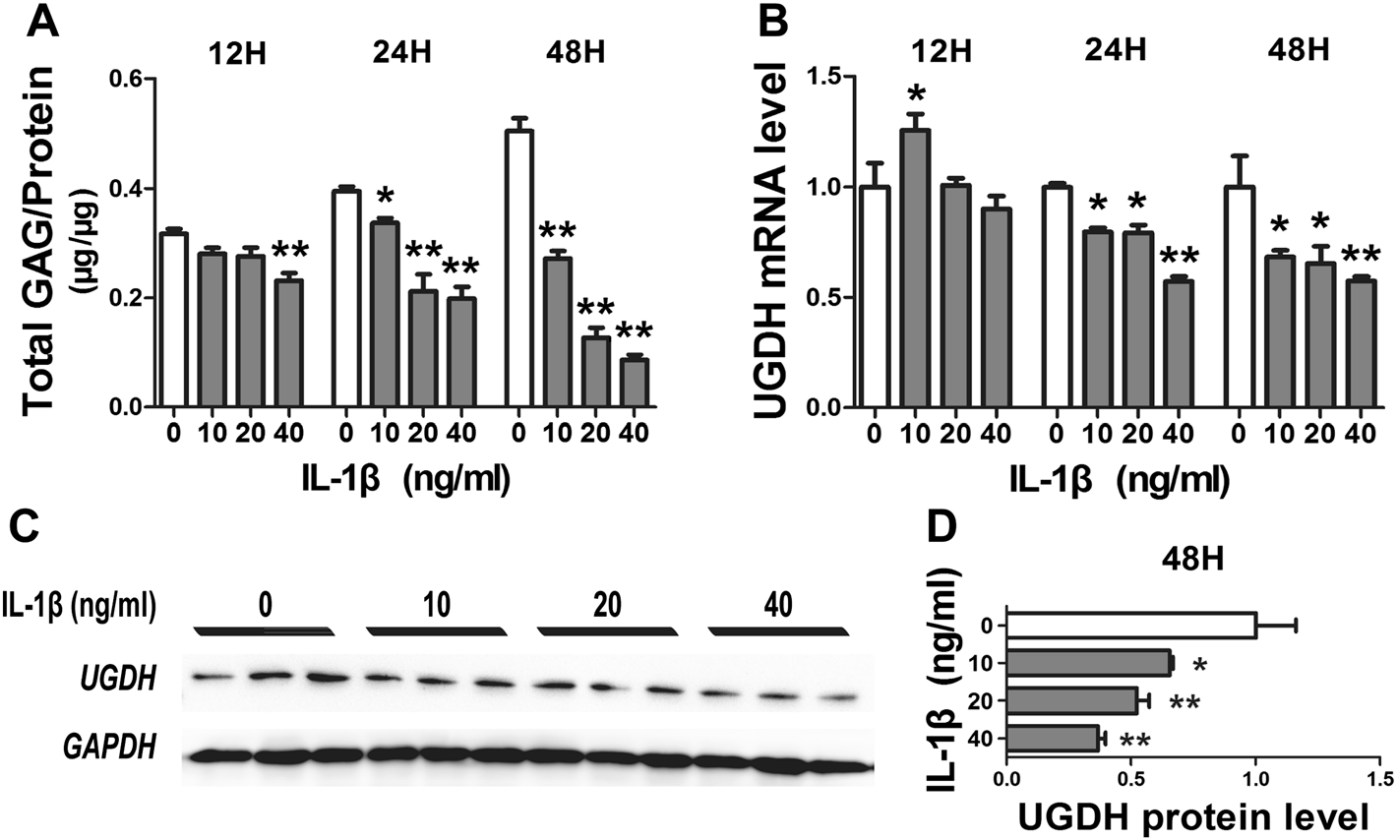
**Interleukin 1β (IL-1β) modulated glycosaminoglycan (GAG) synthesis and UDP-glucose dehydrogenase (** ***UGDH*** **) gene expression.** Human articular chondrocytes were treated with recombinant IL-1β for 12 h, 24 h and 48 h. Then, GAG content **(A)** and the mRNA level of *UGDH* **(B)** of chondrocyte cultures was detected. Meanwhile, UGDH protein level from chondrocytes treated with IL-1β for 48 h was detected using western blotting assay **(C, D)**. Values were presented as mean ± standard error of the mean from at least three independent experiments. *P <0.05 and **P <0.01 versus control.

To determine whether IL-1β was involved in the suppression of UGDH protein expression in OA cartilage, we treated human articular chondrocytes with recombinant IL-1β and found that IL-1β decreased the total GAG content of chondrocyte cultures in a concentration- and time-dependent manner (all *P* <0.05, Figure 3A). Although mRNA level of *UGDH* was increased after 12 h, IL-1β downregulated *UGDH* mRNA level in a concentration-dependent manner after 24 h or 48 h of treatment (*P* <0.05, Figure 2B). Moreover, it also turned out that UGDH protein level was downregulated by IL-1β treatment for 48 h (all *P* <0.05, Figure 3C and D).

### Transcriptional regulation of UGDH

**Figure 4:**
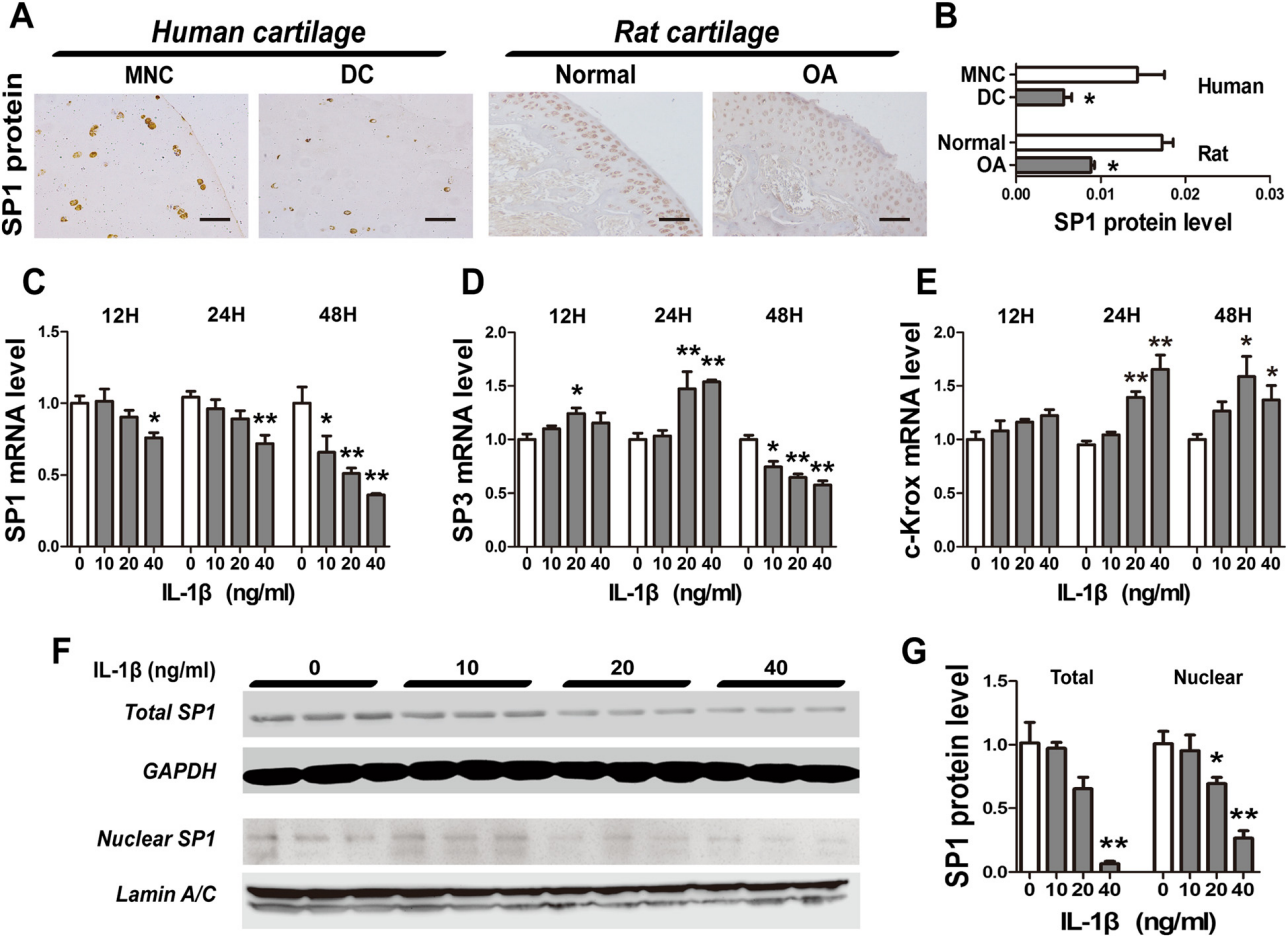
**Gene expressions of trans-regulators of UDP-glucose dehydrogenase (** ***UGDH*** **).** Specificity protein 1 (Sp1) protein level in was detected in both human and rat cartilage samples **(A**, **B)**. Relative mRNA levels of *Sp1*, *Sp3* and Krueppel-related zinc finger protein c-Krox (*c-Krox*) were also detected in human primary chondrocyte treat with IL-1β **(C**-**E)**. Total and nuclear Sp1 protein level was also detected and quantified **(F**, **G)**. MNC, microscopically normal cartilage; DC, degenerative cartilage. Scale bars, 100 μm. Values are presented as mean ± standard error of the mean from at least three independent experiments. *P <0.05 and **P <0.01 versus control.

*Sp1*, *Sp3* and *c-Krox* are the key trans-regulators of the *UGDH* gene [11-13]. Here, *Sp1* protein level in human DC was markedly lower than the MNC of the same patient (*P* <0.05, Figure 4A and B). Meanwhile, a notable decrease of *Sp1* protein level in OA rat cartilage was also observed (*P* <0.05, Figure 4A and B). Moreover, the mRNA expression of *Sp1* in human primary chondrocytes was downregulated after IL-1β treatment, while *c-Krox* mRNA levels were increased (*P* <0.05, Figure 4C and E). *Sp3* mRNA expression was stimulated by IL-1β after 12-h and 24-h treatment, while an obvious decrease in *Sp3* mRNA level was detected after 48 h (*P* <0.05, Figure 4D). A concentration-dependent suppression of protein expression and nuclear translocation of Sp1 were also observed in chondrocytes treated with IL-1β for 48 h (all *P* <0.05, Figure 4F and G). Moreover, the ratio of *Sp3*/*Sp1* and *c-Krox*/*Sp1* was markedly increased after IL-1β treatment (*P* <0.05). An additional figure file shows this in more detail (see Additional file 2).

### IL-1β modulated the expression of *UGDH* through the p38 MAPK and SAP/JNK pathway

**Figure 5:**
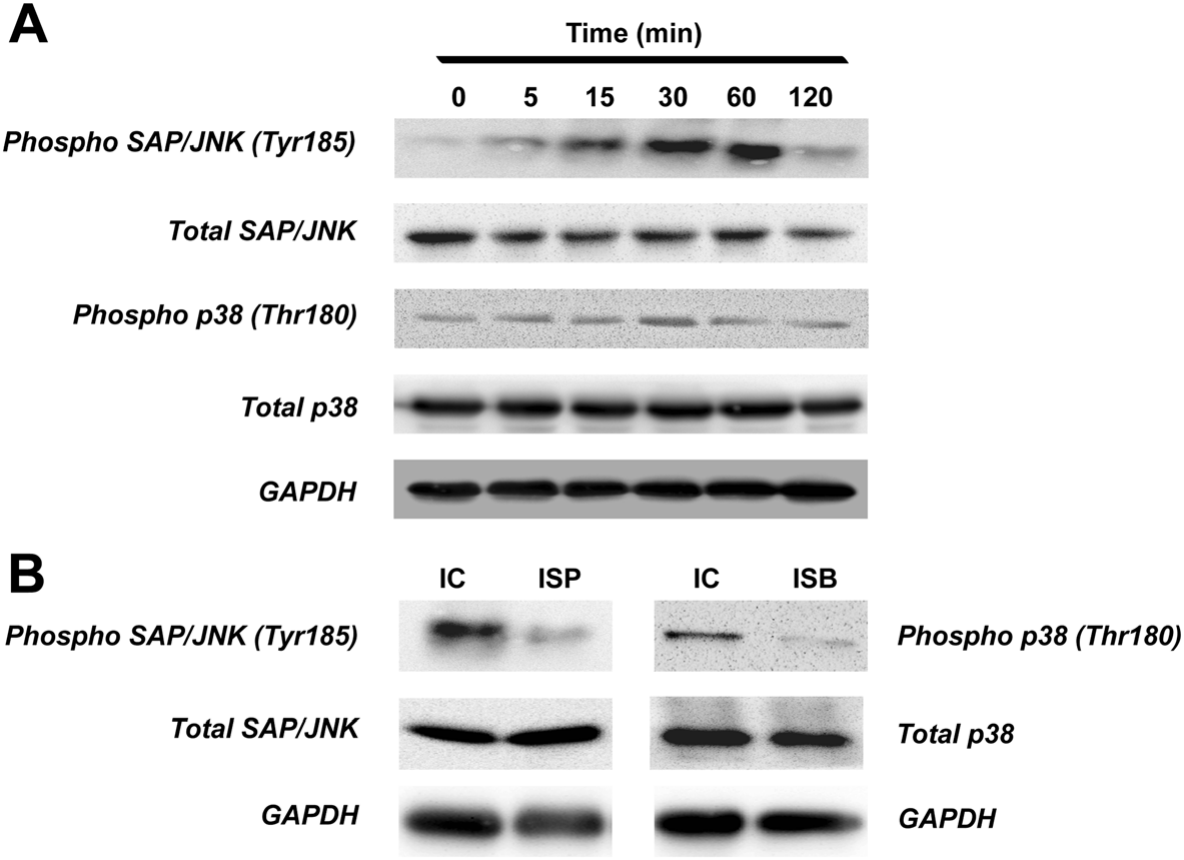
**Phosphorylation of stress-activated protein kinase/c-Jun N-terminal kinas**e **(SAP/JNK) and p38 mitogen-activated protein kinase (MAPK) protein in human primary chondrocytes.** Human primary chondrocytes were treated with IL-1β treatment (10 ng/mL) for 0 to 120 mintes **(A)** or pretreated with SP600125 or SB203580 for 30 minutes and then treated with 10 ng/mL IL-1β for 30 minutes **(B)**. Then, phosphorylated and total protein level of SAP/JNK and p38 MAPK, as well as glyceraldehyde-3-phosphate dehydrogenase (GAPDH) protein level, were detected using western blotting assay. IC, IL-1β control, namely chondrocytes treated with 10 ng/mL IL-1β for 30 minutes; ISP, chondrocytes pretreated with SP600125 for 30 minutes and subsequently co-treated with the SP600125 and 10 ng/mL IL-1β for another 30 minutes; ISB, chondrocytes pretreated with SB203580 for 30 minutes and then co-treated with the SB203580 and 10 ng/mL IL-1β for another 30 minutes.

**Figure 6:**
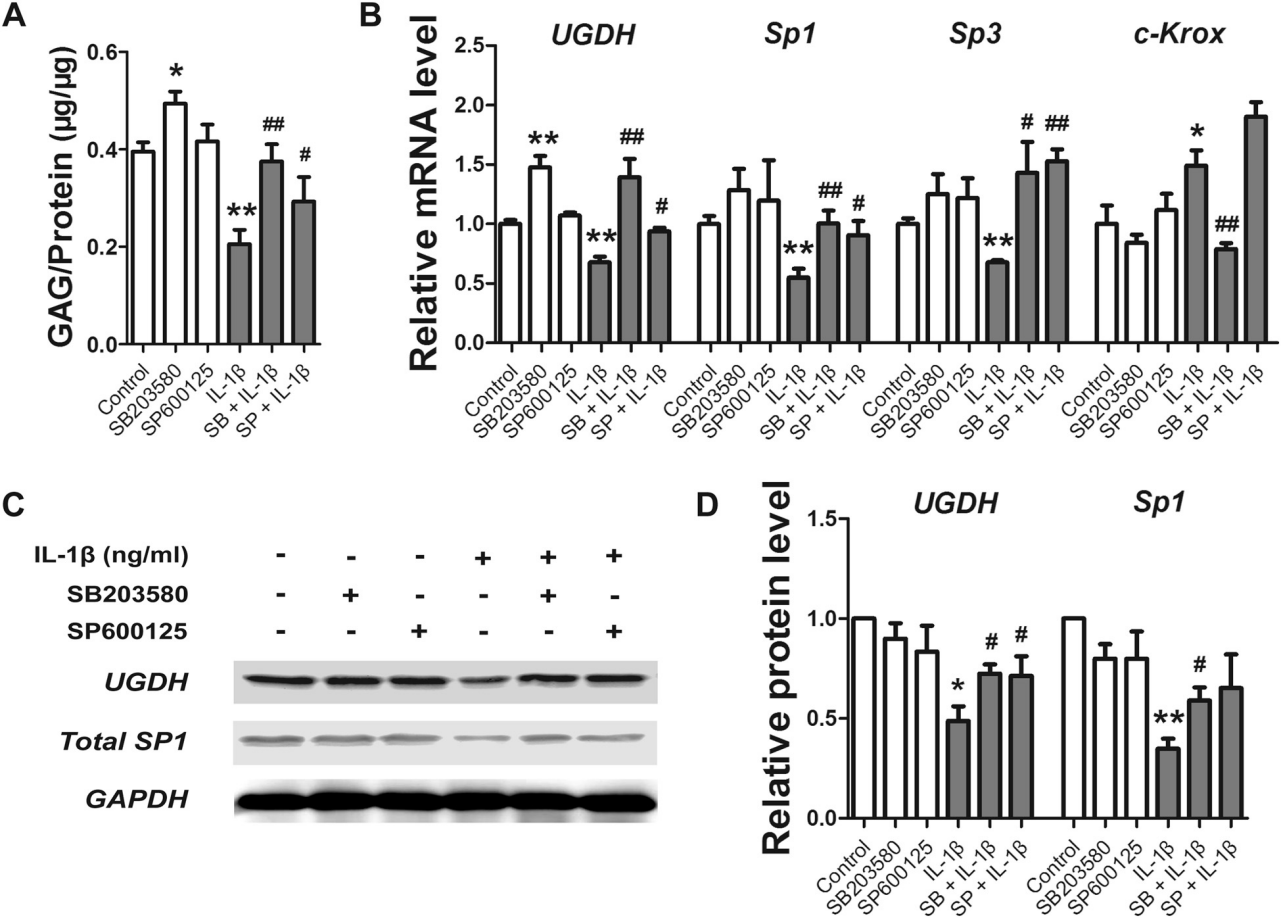
**IL-1β, SB203580 and SP600125 modulated UDP-glucose dehydrogenase (** ***UGDH*** **) gene expression.** Human articular chondrocytes were pretreated with SB203580 (20 μM) and SP600125 (10 μM) for 30 minutes and/or subsequently treated with IL-1β (10 ng/mL) for another 48 h. Total GAG **(A)**, the mRNA level of *UGDH*, specificity protein 1 (*Sp1*), *Sp3* and Krueppel-related zinc finger protein c-Krox (*c-Krox*) **(B)**, and the protein level of UGDH and Sp1 **(C, D)** was also detected. Values are presented as mean ± standard error of the mean from at least three independent experiments. *P <0.05, **P <0.01 versus control, ^#^ *P* <0.05, ^##^ *P* <0.01 versus the IL-1β group.

IL-1β obviously increased the phosphorylation levels of both SAP/JNK and p38 MAPK from 5 minutes to 1 h, or even longer in human articular chondrocytes, while the peak of phosphorylation last from 15 to 60 minutes (Figure 5A). Then, the phosphorylation status of both SAP/JNK and p38 MAPK started to fade after 60 minutes (Figure 5A). However, the activation of phosphorylation of SAP/JNK and p38 MAPK by IL-1β were obviously reduced by the pretreated SP600125 and SB203580, respectively (Figure 5B). Further, SB203580 promoted GAG synthesis and *UGDH* mRNA expression but did not affect the trans-regulators, while SP600125 affected none of these process (*P* <0.05, Figure 6A-D). However, IL-1β inhibited GAG synthesis and gene expression of *UGDH*, *Sp1* and *Sp3*, but stimulated *c-Krox* gene expression (*P* <0.05, Figure 6A-D), while both SB203580 and SP600125 attenuated the effect of IL-1β on these process (*P* <0.05, Figure 6A-D), which indicated that both p38 MAPK pathway and SAP/JNK pathway were involved in the IL-1β-modulated *UGDH* gene expression.

## Discussion

It is well-known that the content of PG is most abundant in the mid-zone of articular cartilage, rather than the superficial or deep zones; chondrocytes in the mid-zone highly synthesize both PGs and collagens, while chondrocytes in the superficial and deep zones mainly synthesize collagens (type II and type X, respectively) instead of PG [24]. Meanwhile, chondrocytes in the mid-zone but not the superficial or deep zones of articular cartilage have high UGDH activity [25], which indicates a possible correlation between UGDH enzyme activity and PG synthesis in articular chondrocytes. Moreover, evidence also indicates that *UGDH* determines hyaluronan synthesis in prostate cancer cells, which thus promotes the metastasis progression of the cancer cells [26], while the stimulated *UGDH* expression by TGF-β promotes hyaluronan production in articular surface cells in chicks [6,7]. In the present study, suppressing *UGDH* gene expression led to an obvious decrease in PG synthesis in human articular chondrocytes. Taken together, these findings suggest that *UGDH* plays a critical role in the PG synthesis of articular chondrocytes, although the intracellular synthesis of UDP-glucuronic acid was not measured in the present study. As PG are the key components in the cartilage matrix, which maintain the fluid and electrolyte balance, and provide the living space of chondrocytes and the elasticity of the cartilage, we speculate that *UGDH* might further be an essential player in maintaining cartilage homeostasis.

As a typical degenerative disease of articular cartilage, OA starts with the disturbance of cartilage homeostasis, which leads to the subsequent loss of cartilage matrix and disorganization of articular cartilage. However, no correlation between the PGs loss and *UGDH* in OA has been reported, except Zemel *et al.* who indicated that no significant increase in UGDH activity was observed between human normal and OA chondrocytes, and that the lack of significantly enhanced UGDH activity could contribute to continuous GAG loss during OA progress [27]. In the present study, we found, for the first time, that protein level of *UGDH* is obviously lower in DC than that of MNC from the same OA patient, while chondrocytes also expressed less *UGDH* protein in rat OA cartilage than that of the normal cartilage. Taken together, the suppressed protein expression and the unchanged enzyme activity of UGDH help to explain the inability of chondrocytes to handle the continuous GAG loss in the advanced OA. However, the OA cartilage samples from either the OA patients undergoing total knee replacement or the rats with papain-induced OA, an aggressive model with an acute local inflammation in the joints and a rapid progress to the terminal stage of OA, were all at their advanced stages, which could not fully replicate the natural pathogenesis of OA dynamically. Other milder models with a more natural and mimic process, like the aging model and running model etc., would be better for the investigation in the role of UGDH in OA. Meanwhile, how the expression of *UGDH* was suppressed in articular chondrocytes still remained unclear.

IL-1β is one of the major pro-inflammatory factors highly expressed in cartilage and synovium throughout the OA pathogenesis and responsible for the PGs loss and cartilage degeneration [8,9,28-30]. However, Maneix *et al.* reported that exogenous IL-1β failed to modulate UGDH enzyme activity in articular chondrocytes [7], while Hickery *et al.* also found that IL-1α, another member of the IL-1 family, could neither modulate UGDH activity [25]. In the present study, we observed that *UGDH* gene expression was stimulated by IL-1β (10 ng/ml) after a 12-hour exposure, which was in accordance with the results from Maneix *et al.* [7], while obvious inhibitions of *UGDH* gene expression were observed after IL-1β treatment at higher concentrations or for longer time, which thus resulted in the suppressed synthesis of GAG in the chondrocytes. All these findings indicated that IL-1β might possibly be involed in the suppression of *UGDH* protein expression in OA cartilage, and that the restricted *UGDH* expression induced by IL-1β, rather than the negligible alteration of UGDH enzyme activity, that might participate in the compensation and decompensation of cartilage matrix during OA pathogenesis. However, as IL-1β presents plentiful effects on cartilage, the functional measurement of IL-1β on GAG-precursor synthesis would further strengthen the evidence in the present study. Meanwhile, as there are multiple factors involved in OA pathogenesis, other stimuli including 17β-oestradiol, TGF-β and IGF-1 could also be involved in this process through modulate either the enzyme activity or gene expression of UGDH [6,7,13,25]. Combining the evidences that UGDH plays an essential role in GAG synthesis and cartilage homeostasis, we suggest that *UGDH* might be possibly a novel target for OA therapy.

Previous studies have demonstrated that IL-1β acts through the activation of downstream signaling cascades. IL-1β binds to type 1 IL-1 receptor (IL-1R1) and then triggers the downstream cascade reaction, which finally leads to the activation of the SAP/JNK, p38 MAPK and NF-κB signaling pathways [10,31,32]. However, although all the three pathways are involved in the metabolic disturbance induced by IL-1β, NF-κB signaling is believed to be mainly responsible for the inflammatory activity of IL-1β. Meanwhile, recent data suggests that it the SAP/JNK and p38 signaling pathway mediatesd the IL-1β-induced suppression of xylosyltransferase I gene expression and the subsequent GAG synthesis in human articular chondrocytes [33]. In the present study, we also observed that inhibition of both p38 MAPK and SAP/JNK led to obvious attenuation of the IL-1β-induced suppression of the gene expression of *UGDH* and its trans-regulators; this indicates that IL-1β could suppress *UGDH* gene expression and consequently inhibit PG synthesis in articular chondrocytes, which might suppress matrix restoration and contribute to the OA progression.

Sp1 binds to the GC or GT rich motifs of *UGDH* promoter sequence and promote transcriptional activity of *UGDH* gene, while Sp3 and c-Krox were suggested to be playing the negative regulatory roles [11-13]. Inhibition of Sp1 expression with siRNA resulted in attenuation of UGDH enzyme activity, reduction of *UGDH* gene promoter activity and consequent depression of *UGDH* mRNA levels [13]. Meanwhile, TGF-β stimulated *UGDH* gene expression through increasing DNA binding of Sp1 to the sequences located in *UGDH* promoter [13]. It was also reported that IL-1β inhibited *COL2A1* gene transcription by increasing the Sp3/Sp1 ratio and inhibiting the binding of Sp1 and Sp3 to the promoter [34]. Binding to the same sequence that binds Sp1 and Sp3, c-Krox was suggested to act in concert with Sp1 and Sp3 to modulate *UGDH* gene expression [11]. Overexpression of c-Krox gene in rabbit articular chondrocytes leads to marked decrease in mRNA and protein level of *UGDH* gene, which is mediated by the increased binding of c-Krox to the cis-sequence located in the *UGDH* promoter [11]. In the present study, IL-1β altered the gene expression of *Sp1*, *Sp3* and *c-Krox*, decreased the nuclear translocation of Sp1 protein, and increased the *Sp3*/*Sp1* ratio, as well as *c-Krox*/*Sp1* ratio. Altogether, it suggests that Sp1, Sp3 and c-Krox mediated the modulation of IL-1β on UGDH gene expression. Sp3/Sp1 ratio and c-Krox/Sp1 ratio in chondrocytes might be helpful in estimating the effects of drugs, cytokines or growth factors on cartilage homeostasis. Moreover, decreasing *Sp3*/*Sp1* and *c-Krox*/*Sp1* ratio could help to restore the cartilage phenotype in osteoarthritic joints.

## Conclusions

In conclusion, *UGDH* plays a critical role in the PG synthesis of articular chondrocytes, of which the expression is suppressed in advanced OA. Meanwhile, IL-1β suppresses *UGDH* gene expression through activating SAP/JNK and p38 MAPK pathways and subsequently modulating the gene expression of *UGDH*’s trans-regulators including Sp1, Sp3 and c-Krox. Accordingly, we speculate that IL-1β might be involved in the suppression of *UGDH* gene expression in OA, which would probably contribute to the OA pathogenesis.

## Additional files

Additional file 1:
**Additional figure that shows relative protein expression of UDP-glucose dehydrogenase (UGDH) and Mankin score of human and rat cartilage.**
Additional file 2:
**Additional figure showing that IL-1β modulated the mRNA expression ratio of specificity protein 3 (** ***Sp3)*** **and** ***Sp1,*** **as well as the ratio of Krueppel-related zinc finger protein c-Krox** **(** ***c-Krox*** **) and** ***Sp1*** **in human primary chondrocytes.**

## Abbreviations

Bp: base pairs
c-Krox: Krueppel-related zinc finger protein cKrox
DC: degenerative cartilage
DMB: 1,9-dimethylmethylene blue
DMEM: Dulbecco’s modified Eagle’s medium
GAG: glycosaminoglycan
GAPDH: glyceraldehyde-3-phosphate dehydrogenase
HE: haematoxylin-eosin
IHC: immunohistochemical
IL-1β: interleukin-1 beta
MNC: microscopically normal cartilage
OA: osteoarthritis
p38 MAPK: p38 mitogen-activated protein kinase
PG: proteoglycans
RT-PCR: reverse transcription polymerase chain reaction
SAP/JNK: stress-activated protein kinase/c-Jun N-terminal kinase
SEM: standard error of the mean
siRNA: small interfering RNA
Sp: specificity protein
TGF-β: transforming growth factor β
UGDH: UDP-glucose dehydrogenase
