# Supplementary material for: UDP-glucose dehydrogenase modulates proteoglycan synthesis in articular chondrocytes: its possible involvement and regulation in osteoarthritis
Source: Arthritis Res Ther. 2014 Dec 3;16(6):484. doi: 10.1186/s13075-014-0484-2 (PMC4298080; doi:10.1186/s13075-014-0484-2)
Supplement: Additional file 1: — Additional figure that shows relative protein expression of UDP-glucose dehydrogenase (UGDH) and Mankin score of human and rat cartilage. [file 13075_2014_484_MOESM1_ESM.pdf]

Additional file 1

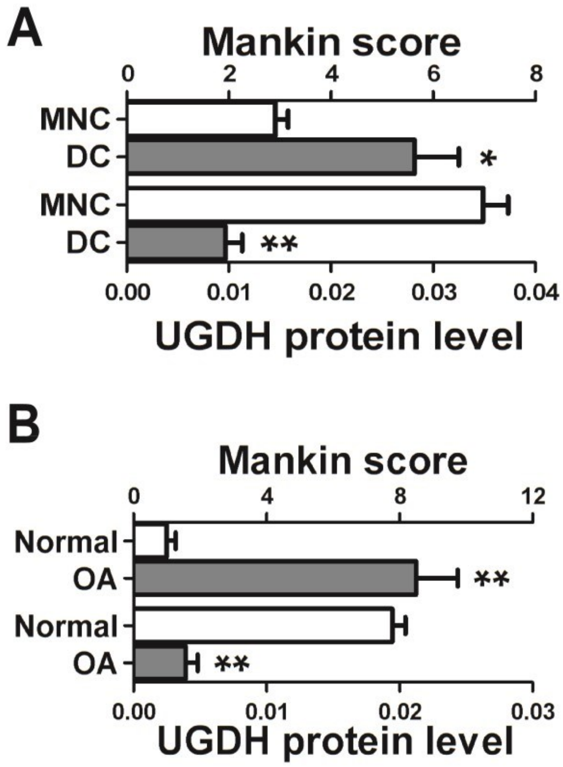

Additional figure 1 UDP-glucose dehydrogenase (UGDH) protein expression and Mankin score of osteoarthritis cartilage. A, UGDH protein level of human cartilage was detected using immunohistochemical (IHC) assay and presented as mean optical density of each chondrocyte using NIS-Elements software (Nikon, Tokyo, Japan). Modified Mankin score system was applied to grade the OA degrees. B, UGDH protein level and Mankin score of rat cartilage were also obtained. Values were presented as Mean ± S.E.M. * $P<0.05$ and * $P<0.01$ *versus* control.
