# Supplementary material for: UDP-glucose dehydrogenase modulates proteoglycan synthesis in articular chondrocytes: its possible involvement and regulation in osteoarthritis
Source: Arthritis Res Ther. 2014 Dec 3;16(6):484. doi: 10.1186/s13075-014-0484-2 (PMC4298080; doi:10.1186/s13075-014-0484-2)
Supplement: Additional file 2: — Additional figure showing that IL-1β modulated the mRNA expression ratio of specificity protein 3 ( Sp3) and Sp1, as well as the ratio of Krueppel-related zinc finger protein c-Krox ( c-Krox ) and Sp1 in human primary chondrocytes. [file 13075_2014_484_MOESM2_ESM.pdf]

Additional file 2

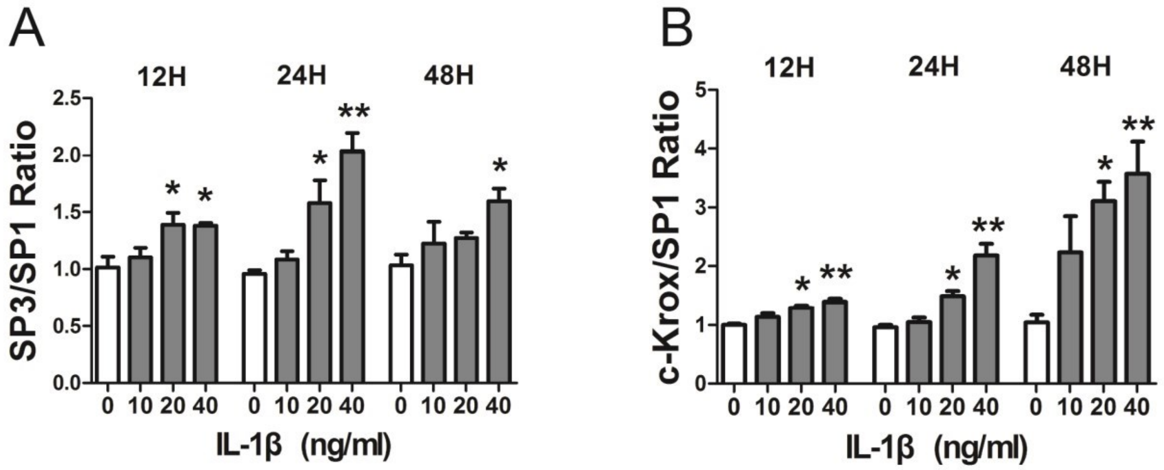

Additional figure 2 Interleukin 1 beta (IL-1β) modulated the ratio of the gene expression of the trans-regulators. Relative mRNA levels of specificity protein 1 (*Sp1*), *Sp3* and Krueppel-related zinc finger protein c-Krox (*c-Krox*) were detected using real time quantitative PCR assay. A, The ratio of Sp3/Sp1 was obtained from the relative mRNA level of *Sp1* and *Sp3*. B, c-Krox/Sp1 ratio was obtained as well. Values were presented as Mean ± S.E.M. from at least three independent experiments. * P<0.05 and ** P<0.01 *versus* control.
